# Association of screen time with parent-reported cognitive delay in preschool children of Kerala, India

**DOI:** 10.1186/s12887-021-02545-y

**Published:** 2021-02-11

**Authors:** Jijo Joseph John, Reny Joseph, Alice David, Ann Bejoy, Kalyan Varghese George, Lisa George

**Affiliations:** 1 Department of Pediatrics, Believer’s Church Medical College, Thiruvalla, Kerala India; 2 MGM Muthoot Hospital, Kozhencherry, Kerala India; 3 Believer’s Church Medical College, Thiruvalla, Kerala India

**Keywords:** Screen use, Preschool children, Cognitive delay, Parental supervision

## Abstract

**Background:**

Screen use is increasing rapidly among preschool children and excess screen use in these children has been associated with cognitive side effects and speech delay. We undertook this study to estimate the risk associated with screen time in children, parental supervision, and parent-reported cognitive development among preschool children aged 2–5 years.

**Methods:**

A cross-sectional study was done between July 2019 and January 2020 involving parents of all students aged 2–5 years, attending 2 kindergarten schools in Thiruvalla using a self-administered questionnaire. Parents also used the Werner David Development pictorial scale (WDDPS), a screening tool to report cognitive development. The schools were sampled based on convenience.

**Results:**

Of the 189 children included in the study, 89.4% had excess screen use (> 1 h per day) and the average use was 2.14 h. 45.0% of parents supervised screen use inconsistently (self-reported). Meal-time screen use (OR 3.8, 95% CI 1.3–10.8), receiving screen on demand (OR 3.7, 95% CI 1.2–11.3), and using devices other than computers (OR 6.5, 95% CI 1.6–26.8) were significantly associated with excess screen use in pre-school children. Similarly, those children with inconsistently supervised screen time were significantly more likely to have suspected deficits in attention (OR 3.2, 95% CI 1.3–8.2), intelligence (OR 4.1, 95% CI 1.3–13.3), and social skills (OR 15.3, 95% CI 1.9–121.2), compared to children whose screen use was consistently supervised.

**Conclusion:**

Screen time in the majority of preschool children is above the recommended limits, and inconsistent supervision by parents was seen in almost half of the study participants. Inconsistently supervised screen time is associated with suspected cognitive delays in children.

**Supplementary Information:**

The online version contains supplementary material available at 10.1186/s12887-021-02545-y.

## Introduction

Digital media and screens are a ubiquitous part of our daily lives and children in today’s world are digital natives. The American Academy of Pediatrics (AAP) and the World Health Organization (WHO) have recommended limiting screen use to less than 1 h in children 2 to 5 years of age, along with an emphasis on selecting high-quality programs, supervised viewing, and co-viewing with the child and avoiding screen time in children younger than 18 months (except for video chatting) [1, 2]. Screen time refers to time spent on any digital media such as television, smartphones, computer, tablets, gaming consoles, etc. India, in the recent past, has seen a major increase in the amount and types of media being consumed. According to a McKinsey report, India is one of the fastest-growing markets for digital consumers, second only to China [3].

The Covid-19 pandemic and the associated lockdown have confined children to their homes and has resulted in an exponential increase in screen use in children [4]. Children under 5 years are a vulnerable population as this is the age where maximum brain growth occurs and increased screen time is associated with adverse neurocognitive outcomes and speech delay [5]. Recent studies with functional cerebral MRI have shown an association between increased screen use and decreased microstructural integrity of brain white matter tracts which supports language and literacy skills [6]. Excess television use in toddlers and preschool children has been demonstrated to have a negative impact on the cognitive, language, and motor skills in this age group [7, 8]. Recent studies with touchscreen devices have also shown excess use in preschool age to be associated with emotional problems, anxiety/depressive symptoms, attention problems, and aggressive behavior, without affecting language delays [9].

Parents are the teachers, facilitators, and gate-keepers of a young child’s media consumption. Screen media parenting practices refer to goal-directed parental interactions with the child, to influence the child’s screen media use [10]. According to a study published by Douglas Gentile et.al, four types of parental monitoring help in healthy screen habits in children – co-viewing with the child, restricting time spent on media, restricting content viewed on-screen, and active mediation- offering opinions on media content, educating the child about the purpose of various media such as advertising and encouraging them to apply practical aspects of the contents viewed to daily life [11].

Only a few studies from India have evaluated screen use in preschool children [12, 13], and as far as we know, no study in India has studied the association of screen time with cognitive delays and parental mediation during screen use. Hence this study was undertaken to estimate the risk associated with screen time in children, parental supervision, and parent-reported cognitive development among preschool children aged 2–5 years.

## Methods

A cross-sectional study was done between July 2019 and January 2020, of parents of all students aged 2–5 years, attending 2 kindergarten schools in Thiruvalla, using a self-administered questionnaire. Parents also used the Werner David Development pictorial scale (WDDPS), a screening tool to assess cognitive development. The schools were sampled based on convenience. These schools are attended by children from upper middle class and middle-class families. Parents who refused to give consent were excluded from the study. Approvals from the Institutional Research Board and Ethics Committee of the hospital were obtained.

### Measures

- Demographics – Information regarding age and sex of the child, parental education and occupation, the pattern of screen use at home, and children’s involvement in other activities were obtained. The socio-economic status of the family was calculated using the modified Kuppuswamy scale, revised for 2017 using real-time update tool, which is a socio-economic scoring scale, validated for use in India [14].
- Parental Supervision– Parental practices regarding the child’s screen viewing, such as restricting screen time, supervising content, and co-viewing with the child, were obtained using a self-reported questionnaire. Co-viewing (shared screen), implies interacting with the child and asking questions related to the content viewed [1]. Parents responded to “how often do you supervise your child’s screen use” as all the time (1), some of the time (2), and never (3). Self-reported parental supervision was categorized as consistent and inconsistent, with responses 2 and 3 being considered inconsistent.
- Cognitive development: Parents were asked to fill the WDDPS scale [Appendix- supplementary file 1). WDDPS is taken from the book by David Werner titled ‘ Disabled Village Children- a guide for community health workers, rehabilitation workers and families’, published by the Hesperian Foundation and adapted for India by the Voluntary Health Association of India. This chart has also been mentioned in the book entitled ‘Family care for children with disabilities- Practical guidance for frontline workers in Low and Middle-income countries’, which has been endorsed by USAID, World Learning and Partnership for every child [15]. There are two charts, one representing physical development and one representing mental and social development. We used the mental and social development chart, which is a screening tool, with age-appropriate pictorial representation of cognitive milestones. This scale assessed 6 components (domains) such as communication, social skills, self-care, attention span, play, and intelligence based on the achievement of an essential skill per domain, at ages of 3 months, 6 months, 9 months, 1 year, 2 years, 3 years and 5 years [16]. Children who had not achieved the age-appropriate milestone were considered to have a suspected delay. We also asked parents about their perception of speech delay and social interaction in their child.

Printed questionnaires were sent home with the students along with an information sheet about the study and consent form, and completed response sheets were collected back from the students after 1 week. Parents filled the questionnaires at home.

### Statistical methods

Descriptive statistics comprising of frequencies and percentages were calculated. Proportions were compared using Fisher’s exact test. Estimates of risk were calculated using Odds Ratio with 95% confidence interval, where the response variables were ‘excess screen time’ or ‘inconsistent supervision’ depending on the question. The explanatory variables include all determinants and social and development related factors that were significantly different. A logistic regression model was built with the determinants alone. Data was analyzed using SAS University Edition.

## Results

Of all the 240 children surveyed, only 189 (79%) were included in the final analysis. Reasons for exclusion include, below 2 years of age (7%), lack of consent (8.3%), and incomplete forms (10%). Among the ones included, 89.9% viewed some form of screen more than the recommended 1 h per day limit. The mean + SD being 2.1 h + 0.4 h.

Demographic data for the study sample are included in Table 1. Screen time was not associated with the child’s gender (*p* = 0.91), mother’s education (*p* = 0.16), or with the mother’s occupation (*p* = 0.32). Similarly, supervised screen viewing was not significantly different for gender (*p* = 0.69), the mother’s education (*p* = 0.18), or the mother’s occupation (*p* = 0.50). Every child in our study belonged to an upper middle-class family.

**Table 1:** Socio demographic characteristics associated with screen time and supervision

Characteristics		Total (*N* = 189)	Screen time			Supervised screen viewing		
			> 1 Hr (*N* = 170)	< 1 Hr (*N* = 19)	*p* value	Inconsistent (*N* = 85)	Consistent (*N* = 104)	*p* value
		n (%)	n (%)	n (%)		n (%)	n (%)	
Child	Female	92 (48.7)	83 (48.8)	9 (47.4)	0.91	40 (47.1)	52 (50.0)	0.69
Mother	Post Graduate	79 (41.8)	73 (42.9)	5 (26.3)	0.16	31 (36.5)	48 (46.2)	0.18
	Housewife	69 (36.7)	64 (37.9)	5 (26.3)	0.32	33 (39.3)	36 (34.6)	0.5

We considered various factors as potential determinants of screen time and supervised screen viewing, including age at introduction, ownership of the device, types of screen, content viewed, time of use, the context of screen used, and forms of supervision as seen in Tables 2 and 3. Risk estimates of those factors that were found to be significant are given in Table 4. Factors that were significantly associated with excess screen time include viewing during mealtime (*p* = 0.01) and allowing the child to use a screen whenever the child demanded (*p* = 0.01), with an estimated risk of 3.4 (OR 3.4, 95% CI 1.3–8.9), and 3.7 (OR 3.7, 95% CI 1.3–10.8) times respectively. Using the screen only up to the recommended time of 1 h per day was associated with viewing on a computer (*p* = 0.05), planning the time when the child could view (*p* = 0.04), and co-viewing (*p* = 0.01). On adjusting for other factors, not using computers, mealtime viewing, and on-demand viewing emerged as independent risk factors of excess screen time with a risk estimate of 6.5 (OR 6.5, 95% CI 1.6–26.8), 3.8 (OR 3.8, 95% CI 1.3–10.8) and 3.7 (OR 3.7, 95% CI 1.2–11.3) times respectively.

**Table 2:** Potential determinants of screen time & parental supervised screen viewing

Characteristics		Total (*N* = 189)	Screen time			Supervised screen viewing		
			> 1 Hr (*N* = 170)	< 1 Hr (*N* = 19)	*p* value	Inconsistent (*N* = 85)	Consistent (*N* = 104)	*p* value
		n (%)	n (%)	n (%)		n (%)	n (%)	
Age at Introduction	< 1 year	13 (6.9)	13 (7.7)	0 (0.0)	0.37	6 (7.1)	7 (6.7)	1
Own	Own Device	14 (7.4)	12 (7.1)	2 (10.5)	0.64	10 (11.8)	4 (3.9)	0.05*
Type of Screen	TV	145 (76.7)	131 (77.1)	14 (73.7)	0.78	63 (74.1)	82 (78.9)	0.49
	Mobile	98 (51.9)	90 (52.9)	8 (42.1)	0.47	48 (56.5)	50 (48.1)	0.31
	Tablet	15 (7.9)	12 (7.1)	3 (15.8)	0.18	6 (7.1)	9 (8.7)	0.79
	Video Game	2 (1.1)	2 (1.2)	0 (0.0)	1.00	0 (0.0)	2 (1.9)	0.50
	Computer	15 (7.9)	11 (6.5)	4 (21.1)	0.05*	3 (3.5)	12 (11.5)	0.06
Content Viewed	Gaming	26 (13.8)	24 (14.1)	2 (10.5)	1.00	11 (12.9)	15 (14.4)	0.83
	Cartoon	156 (82.5)	142 (83.5)	14 (73.7)	0.34	66 (77.7)	90 (86.5)	0.13
	YouTube	49 (25.9)	42 (24.7)	7 (36.8)	0.27	23 (27.1)	26 (25.0)	0.87
	Other Devices	14 (7.4)	12 (7.1)	2 (10.5)	0.64	5 (5.9)	9 (8.7)	0.58
Time of Use	< 1Hr Before Bed	112 (59.3)	99 (58.2)	13 (68.4)	0.47	53 (62.4)	59 (56.7)	0.46
	Meal Time	137 (72.5)	128 (75.3)	9 (47.4)	0.01*	56 (65.9)	81 (77.9)	0.07
Context of Screen Use	On Demand	102 (54.0)	97 (57.1)	5 (26.3)	0.01*	53 (62.4)	49 (47.1)	0.04*
	Reward	32 (16.9)	29 (17.1)	3 (15.8)	1.00	12 (14.1)	20 (19.2)	0.44
	Pacify	26 (13.8)	25 (14.7)	1 (5.3)	0.48	14 (16.5)	12 (11.5)	0.40
	Tantrums	110 (58.2)	101 (59.4)	9 (47.4)	0.34	50 (58.8)	60 (57.7)	0.88
	Planned	41 (21.7)	33 (19.4)	8 (42.1)	0.04*	11 (12.9)	30 (28.9)	0.01*
Supervision	Consistent Supervision	104 (55.0)	95 (55.9)	9 (47.4)	0.63	N/A	N/A	N/A
	Co-view	136 (72.0)	44 (25.9)	9 (47.4)	0.06			
	Monitor - Content	176 (93.1)	159 (93.5)	17 (89.5)	0.62			
	Monitor - Time	184 (97.4)	166 (97.7)	18 (94.7)	0.41			
	Set Time Limit	135 (71.4)	128 (75.3)	19 (100.0)	0.01*			
	Screen Free Interval	137 (72.5)	125 (73.5)	12 (63.2)	0.42			
Screen Time	> 1 Hour	170 (89.9)	N/A	N/A	N/A	75 (88.24)	95 (91.35)	0.63

*p < 0.05

**Table 3:** Cognitive development - screen time & parental supervision

Characteristics		Total (*N* = 189)	Excess screen time			Supervised screen viewing		
			> 1 Hr (*N* = 170)	< 1 Hr (*N* = 19)	*p* value	Inconsistent (*N* = 85)	Consistent (*N* = 104)	*p* value
		n (%)	n (%)	n (%)		n (%)	n (%)	
Activity	Physical Activity > 1 Hr	122 (64.6)	108 (63.5)	14 (73.7)	0.46	46 (54.1)	76 (73.1)	0.01*
	Draw	126 (66.7)	113 (66.5)	13 (68.4)	1.00	50 (58.8)	76 (73.1)	0.04*
	Craft	54 (28.6)	49 (28.8)	5 (26.3)	1.00	24 (28.2)	30 (28.9)	1.00
	Interact – Playmates	134 (70.9)	123 (72.4)	11 (57.9)	0.19	63 (74.1)	71 (68.3)	0.42
	Interact - Older Family	86 (45.5)	80 (47.1)	6 (31.6)	0.23	37 (43.5)	49 (47.1)	0.66
	Other Activity	32 (16.9)	29 (17.1)	3 (15.8)	1.00	8 (9.4)	24 (23.1)	0.02*
Suspected Cognitive Delay as per WDDPS	Communication	18 (9.5)	17 (10.0)	1 (5.3)	1.00	10 (11.8)	8 (7.7)	0.46
	Social Interaction	12 (6.3)	10 (5.9)	2 (10.5)	0.34	11 (12.9)	1 (1.0)	< 0.005*
	Self-Care	7 (3.7)	6 (3.5)	1 (5.3)	0.53	3 (3.5)	4 (3.9)	1.00
	Attention	23 (12.2)	21 (12.4)	2 (10.5)	1.00	16 (18.8)	7 (6.7)	0.01*
	Play	13 (6.9)	12 (7.1)	1 (5.3)	1.00	9 (10.6)	4 (3.9)	0.09
	Intelligence	16 (8.5)	14 (8.2)	2 (10.5)	0.67	12 (14.1)	4 (3.9)	0.02*

*p < 0.05

**Table 4:** Risk estimates of potential determinants of screen time & supervision

Characteristics	Excess screen time		Inconsistent supervision	
	OR (95% CI)	Adj. OR (95% CI)	OR (95% CI)	Adj. OR (95% CI)
Own Device	1.5 (0.3–7.5)	NS	0.3 (0.09–1.0)	NS
Not Computer	3.9 (1.1–13.1)*	6.5 (1.6–26.8)*	3.6 (1.0–13.1)	5.1 (1.3–19.6)*
Meal Time	3.4 (1.3–8.9)*	3.8 (1.3–10.8)*	0.6 (0.3–1.0)	0.5 (0.2–1.0)*
On Demand	3.7 (1.3–10.8)*	3.7 (1.2–11.3)*	1.9 (1.0–3.3)	NS
Not Planned	3.0 (1.1–8.1)*	NS	2.7 (1.3–5.8)	2.6 (1.1–6.0)*
No Time limit	N/A	NS	2.1 (1.1–4.3)	NS
Not Co-view	2.6 (1.0–6.8)*	NS	7.3 (3.2–16.7)	7.8 (3.3–18.2)*

*NS* not significant, *N/A* not applicable

* *p* < 0.05

Inconsistent supervision was associated with the child having a device of his or her own (*p* = 0.05), when viewing on devices other than a computer (*p* = 0.06), during mealtime (*p* = 0.07), when permitted to view when the child demanded it (*p* = 0.01), when viewing was not planned (*p* = 0.01), when no time limit was set (*p* = 0.02) and when co-viewing was not involved (*p* = 0.01). However on adjusting for all significant factors, only using screen devices other than a computer, unplanned viewing and lack of co-viewing emerged as independent risk factors for inconsistent supervision, with an estimated risk of 5.1 (OR 5.1, 95% CI 1.3–19.6), 2.6 (OR 2.6, 95% CI 1.1–6.0) and 7.8 (OR 7.8, 95% CI 3.3–18.2) times respectively.

We also considered various factors that could potentially be the effect of excess screen time or inconsistent parental supervision while viewing, including various activities and developmental markers as per WDDPS. Risk estimates of potential effects of excess screen time and inconsistent supervision are given in Table 5. While none of the factors were associated with excess screen time, almost all of them were associated with inconsistent parental supervision. Inconsistent supervision is associated with children being involved in physical activity for less than 1 h per day (*p* = 0.01), not drawing (*p* = 0.04) and not being interested in other activities (*p* = 0.02) with a risk estimate of 2.3(OR 2.3, 95% CI 1.3–4.2), 1.9 (OR 1.9, 95% CI 1.0–3.5), and 2.9(OR 2.9, 95% CI 1.2–6.8) times respectively.

**Table 5:** Risk estimates of potential effects of screen time & supervision

Characteristics		Excess screen time	Inconsistent supervision
		OR (95% CI)	OR (95% CI)
Activities	Physical Activity < 1 Hr	1.6 (0.6–4.7)	2.3 (1.3–4.2)*
	Not Drawing	1.1 (0.4–3.0)	1.9 (1.0–3.5)*
	No other activities	0.9 (0.3–3.3)	2.9 (1.2–6.8)*
Cognitive Delay as per WDDPS	Social Interaction	0.5 (0.1–2.6)	15.3 (1.9–121.2)*
	Attention	1.2 (0.3–5.6)	3.2 (1.3–8.2)*
	Intelligence	0.8 (0.2–3.6)	4.1 (1.3–13.3)*

*p < 0.05

Moreover, inconsistent supervised viewing is associated with suspected cognitive delays, as per WDDPS, such as delay in attention (*p* = 0.01), intelligence (*p* = 0.02), and in social interactions (*p* < 0.005), with a risk estimate of 3.2 (OR 3.2, 95% CI 1.3–8.2), 4.1 (OR 4.1, 95% CI 1.3–13.3) and 15 (OR 15.3, 95% CI 1.9–121.2) times when compared to children whose screen use are consistently supervised respectively.

## Discussion

Early childhood is a period of significant cognitive and behavioral development. Cognitive skills that help children to understand and process information, interact with their environment, and use logic to understand mathematical and scientific processes are garnered during this time [17]. Much like other habits, viewing habits that begin in infancy persist into childhood [18]. Pediatric societies all over the world recommend limiting screen use among preschool children and emphasize on consistent parental mediation to offset the harmful effects of screen. However, adherence to these guidelines is poor and shows a diminishing trend [1, 19, 20]. This study builds on previous research, evaluating the determinants of excess screen use and inconsistent parental supervision of screen viewing and its association with suspected cognitive delays among young children in the Indian context.

According to 2 other studies from India, less than 20% of preschool children use a screen for less than 1 h a day [12, 13]. Our study demonstrated similarly, with an average screen use of 2.1 h per day and 89.9% exceeding the AAP recommendations. Similar to most published literature, we found that the commonest screen used was TV (76.7%), and the type of content most watched were cartoons (82.5%) [12, 21]. We found children who used devices other than a computer were 6.5 times more likely to have excess screen time. Also, children who did not have a structured screen use plan and a set time limit were three to four times more likely to have excess screen time. Most parents provided screen time on demand (54.0%) and during mealtimes (72.5%). Irrespective of other factors, these two factors were significantly associated with excess screen use. Interestingly other studies have reported a much lower prevalence of meal-time screen use (22–50%) [22, 23], and most pediatric societies recommend avoiding screen use during meal times [1, 24]. A recently published study from Lithuania also found a similar significant association between mealtime screen use and excess screen use among children aged 2–5 years [22].

Our study had very few children (6.9%) who started viewing before the age of 1 year, unlike reported literature which has shown that significant media exposure starts from around 6 months of age [25]. It has been shown that children comprehend child-directed television only from around 2 years of age [22]. Exposure to adult-directed television early in life, as well as high background television exposure, has negative correlations with the child’s executive functioning and cognitive development [26]. Similar effects are not seen when infants viewed child-oriented media [26].

The cognitive impact of screen use on the very young child depends not only on the quantity and the social context of media used but also on the quality of programs [25]. Active media (video games/completing homework on the computer) have been shown to improve academic performance and reduce school absenteeism [27]. Passive screen time involving receiving passive screen-based information is harmful especially for preschool children, except for certain age-appropriate programs such as ‘Sesame Street’ [28]. It is of concern that active screen use was seen in only about 13% of the children in our study and cartoon viewing was almost ubiquitous. Moreover, the pacing of content in shows designed for children is extremely rapid. This auditory and visual over-stimulation may prime children to expect similar fast-paced activities in daily life, and the lack thereof can lead to inattention [29, 30].

Parent-reported suspected cognitive delay measured using the WDDPS was not found to be associated with excess screen use. This is probably because of the simplicity of the screening tool used, the number of children with suspected cognitive delay being low and the majority of the children (89.9%) having excess screen time. Various studies have shown a strong association between increased television use (> 2 h per day), and significant language delays [19, 28, 31]. In a study from Taiwan, Lin et.al demonstrated a significantly higher cognitive delay among children with increased television exposure (OR 3.9, 95% CI 1.4–5.9) [7]. In contrast to our study, a recent systematic review and meta-analysis revealed that children with excess screen time were at a higher risk of delayed language development, learning problems (language and mathematics), and reading problems [32].

Apart from screen time, we also studied the four types of parental supervision, since supervised viewing and co-viewing has also been recommended by AAP, including active supervision, co-viewing, restricting time spent on screen, and restricting the content [10, 11]. In our study, while 97.4% of the parents employed restrictive supervision and set rules regarding screen time and content, only about 28% of parents co-viewed media content with their child. Moreover, only 55% were able to consistently supervise children during screen use. We examined if the same factors that determine screen time would also determine supervision. In the univariate analysis, children who had their own device, who used screens other than a computer, got screen on demand, and had unplanned screen time were significantly more likely to have inconsistent parental supervision. These factors are similar to the ones that determine excess screen time. Independent factors include using devices other than a computer, not having a structured plan, and not co-viewing.

There is growing evidence to suggest that children learn the most from screens when caregivers are actively engaged in viewing content with them [33]. Suspected cognitive delays were reported by parents using WDDPS among those who had inconsistent supervision. These children were 15.3 times more likely to have a delay in social interactions, 3.2 times more likely to have attention deficit, and 4.1 times more likely to have intelligence delay.

We also considered the association of inconsistent supervision on routine activities and found that inconsistently supervised children were 2.3 times more likely to have less than 1 h of physical activity, 1.9 times more likely not to draw, and 2.9 times more likely not to be interested in other activities. Though a cross-sectional study of more than 8000 US children did not find any significant association between screen time and physical activity [34], data regarding parental supervision during screen use and correlation with physical activity, and cognitive delay was lacking.

There are certain limitations to our study. Attitude and determinants of parental screen time and effect of sibling screen use were not assessed. Cognitive development and speech delay was assessed based on a parental questionnaire and a formal assessment was not done. The scale used to assess cognitive development was not validated for the sample studied. The use of a parental questionnaire may have led to recall bias and the veracity of the responses could not be ascertained. Also as the children included in our study were all from the upper middle class, the results might not be generalizable to the entire population. Moreover, the lack of association of excess screen time with various factors studied may be due to the low prevalence of those who watch less than 1 h a day. This study looked at associations between screen use and cognitive delays and direct causal relationships have not been ascertained.

This study throws light on the importance of consistent parental supervision, avoidance of screen during meal times, and a structured media plan for preschool children. Larger population-based surveys are the need of the hour to understand the effects of this growing pandemic on this vulnerable population.

## Conclusions

Screen use in the sample population is much higher than the recommended daily limit of 1 h per day. Avoiding screen use with meal times and giving screen to the child only in a planned manner are practical tips to prevent excess screen use in children. Restricting screen use to computers may give more control for the parents to supervise screen use in children. Inconsistent parental supervision is suspected to affect cognitive outcomes of preschool children.

## Supplementary Information

**Additional file 1.** Questionnaire used in the study including Werner David Developmental Pictorial Scale (WDDPS).

## Data Availability

The dataset used/analyzed during the current study is available from the corresponding author on reasonable request.

## Abbreviations

WDDPS: Werner David Developmental Pictorial Scale
AAP: American Academy of Pediatrics
WHO: World Health Organization
MRI: Magnetic resonance imaging
KG: Kindergarten
SD: Standard deviation
