# Supplementary material for: Association of screen time with parent-reported cognitive delay in preschool children of Kerala, India
Source: BMC Pediatr. 2021 Feb 11;21:73. doi: 10.1186/s12887-021-02545-y (PMC7875762; doi:10.1186/s12887-021-02545-y)
Supplement: Supplementary file 1 — Additional file 1. Questionnaire used in the study including Werner David Developmental Pictorial Scale (WDDPS). [file 12887_2021_2545_MOESM1_ESM.docx]

**SCREEN TIME QUESTIONNAIRE**

*(Please tick against your answer)*

1. Name of Child: ………………………………………………………………………………………………..
2. Name of School: ……………………………………………………………………………………………..
3. Father’s education and occupation: ……………………………………………………………………
4. Mother’s education and occupation: ……………………………………………………………………
5. Who takes care of the child at home
   - Parent
   - Grandparent
   - Maid
   - Older sibling
6. Number of siblings
   - 1
   - 2
   - 3
   - 4
   - Others ………………………………………
7. Order of the child in question
   - 1
   - 2
   - 3
   - 4
   - Others ……………………………………….
8. Age of child:

- 0-18 months
- 18-24 months
- 2-5 years

1. What is the average time your child spends on the screen?

- <1 hour ( ………. Mins)
- 1-3 hours (………. Hours)
- 3-5 hours (………… Hours)
- 5 hours (…………Hours)

1. Which type of device does your child use?

- TV
- Mobile
- Tablet
- Video Games
- Computer/Laptop

1. Do you supervise your child when he uses devices?

- All of the time
- Some of the time
- Never

1. What is the content that your child sees on the devices?

- Gaming
- Cartoons/Movies
- YouTube
- Others, specify………………….

1. At what age was your child introduced to screens/devices?

- Between 0- 12 months
- Between 1-3 years
- Between 3-5 years

1. In what context does your child use the screen?

- On demand
- As a reward
- To pacify the child in stressful conditions
- Others, specify…………….

1. Is the content that your child sees on the screen monitored?

- Yes
- No

1. Do you co-view with your child as he/she uses the digital device?

- All the time
- Some of the time
- Never

11. Does your child have his/her own device?

- Yes
- No

12. Do you set a daily time limit for your child’s screen time?

- Yes
- No

13. Do you impose screen free time intervals/ screen free areas ?

- Yes
- No

14. How many hours does your child engage in physical activity?

- < 30 mins
- 30 mins – 1 hour
- 1-3 hours
- > 3 hours

15. Does your child show interest in activities other than digital devices?

- Playing with friends
- Drawing and colouring
- Arts and craft
- Spending time with older relatives
- Others, specify………………

16. Does your child show interest in interacting with children who are his/her age?

- Yes
- No

17. How many hours before bedtime is the digital device taken from the child?

- 1 hour
- 2 hours
- 3 hours
- More than 3 hours
- Child goes off to sleep while watching the digital device

18. Do you feel that your child has speech delay for his age?

- Yes
- No

19. Are you able to control your child’s screen time?

- Yes
- No

20. Does your child use any device during meal times?

- All of the time
- Some of the time
- Never

21. Does your child throw tantrums if refused any devices?

- All of the time
- Some of the time
- Never


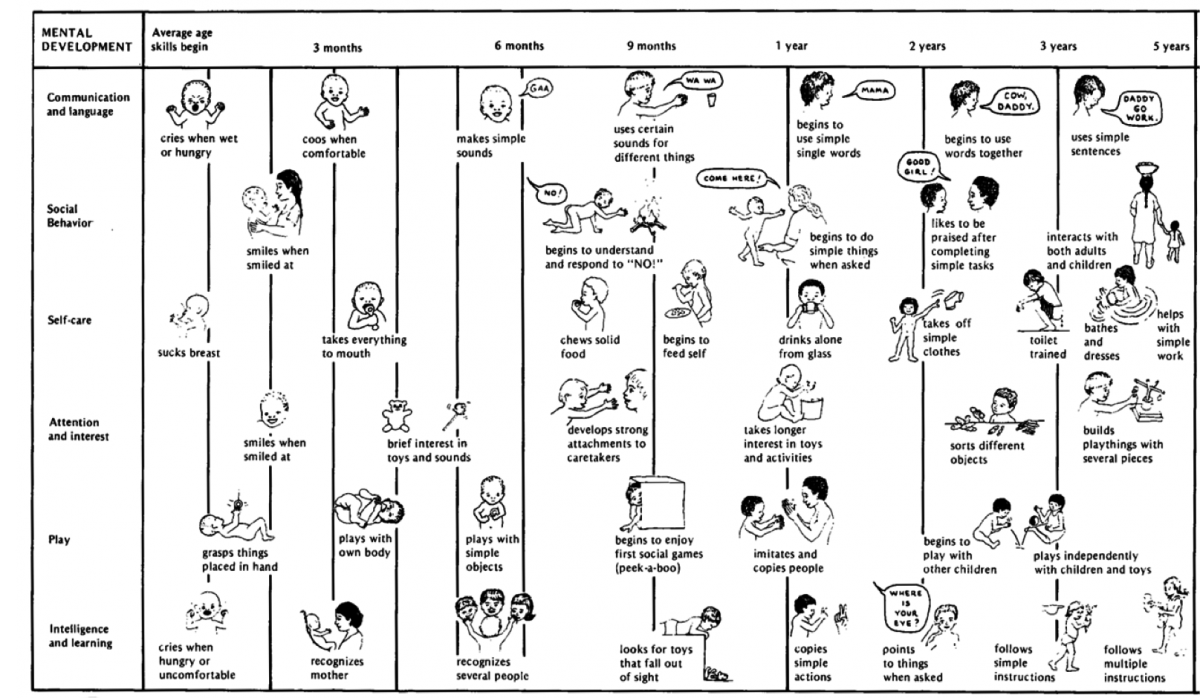


KINDLY MARK ON THE CHART WHAT YOUR CHILD IS CURRENTLY ABLE TO DO
